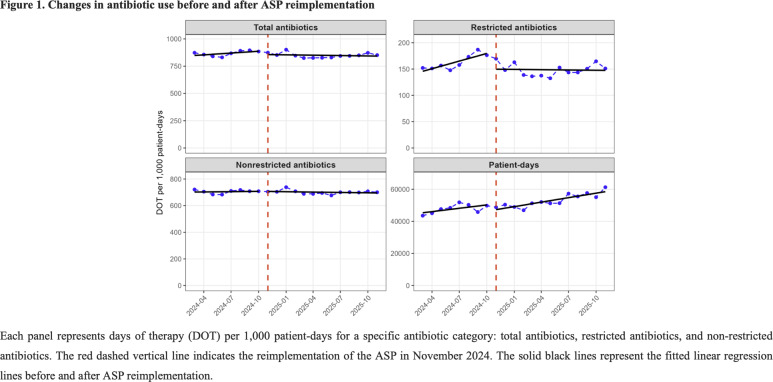# 75 Antibiotic Susceptibility Patterns of Extended-Spectrum Beta-Lactamase-Producing Enterobacterales (ESBL-E), Tennessee 2019–2024

**DOI:** 10.1017/ash.2026.10503

**Published:** 2026-06-23

**Authors:** Wongu Kang, Yeonjin Son, Jiae Kim, Kyungkeun Cho, Inah Park, So Yun Lim, Seongman Bae, Sung-Han Kim

**Affiliations:** 1 Asan medical center; 2 Asan Medical Center; 3 Office for Infection Control, Asan Medical Center, Seoul, Korea, Department of Infectious Diseases, Asan Medical Center, University of Ulsan College of Medicine, Seoul, Korea

## Abstract

**Background:** In early 2024, a nationwide healthcare system disruption in South Korea led to temporary suspension of the antimicrobial stewardship program (ASP) at a tertiary care center due to critical workforce shortages, which was accompanied by increased use of antibiotics, particularly restricted agents. In November 2024, a national ASP pilot program enabled reimplementation of ASP activities. We evaluated changes in hospital-wide antibiotic consumption following ASP reimplementation. Methods This retrospective, single-center study evaluated hospital-wide antibiotic use from March 2024 to November 2025 at a 2,446-bed tertiary care center (Asan Medical Center, Seoul). Antibiotics were classified as restricted agents subject to ASP interventions (preauthorization and prospective audit and feedback) and nonrestricted agents without such interventions. Changes in monthly days of therapy (DOT) per 1,000 patient-days were analyzed using interrupted time-series analysis, with November 2024 defined as the intervention time point. Results Over a 21-month study period, a total of 1,070,544 patient-days were included. Following reimplementation of the ASP, interrupted time-series analyses showed a reduction in total antibiotic use, corresponding to a 7.9% relative decrease (95% CI, 12.6% to −2.2%). This overall change was driven primarily by restricted antibiotics, which demonstrated significant reductions in both level (−29.8 DOT per 1,000 patient-days; 95% CI, 50.6 to −9.05; P = .012) and trend (−5.03 DOT per 1,000 patient-days per month; 95% CI, −7.65 to −2.41; P = .0015), yielding a 29.7% relative reduction (95% CI, −36.0% to −21.8%) compared with projected counterfactual use. In contrast, nonrestricted antibiotics showed no statistically significant changes. Conclusion Although reductions in restricted antibiotic use following ASP implementation are well recognized, our findings demonstrate that stewardship effects can be effectively restored after temporary program interruption. This resilience underscores the role of antimicrobial stewardship as a system-level safeguard against inappropriate antibiotic escalation, particularly in the context of healthcare system disruptions.

**Figure:**
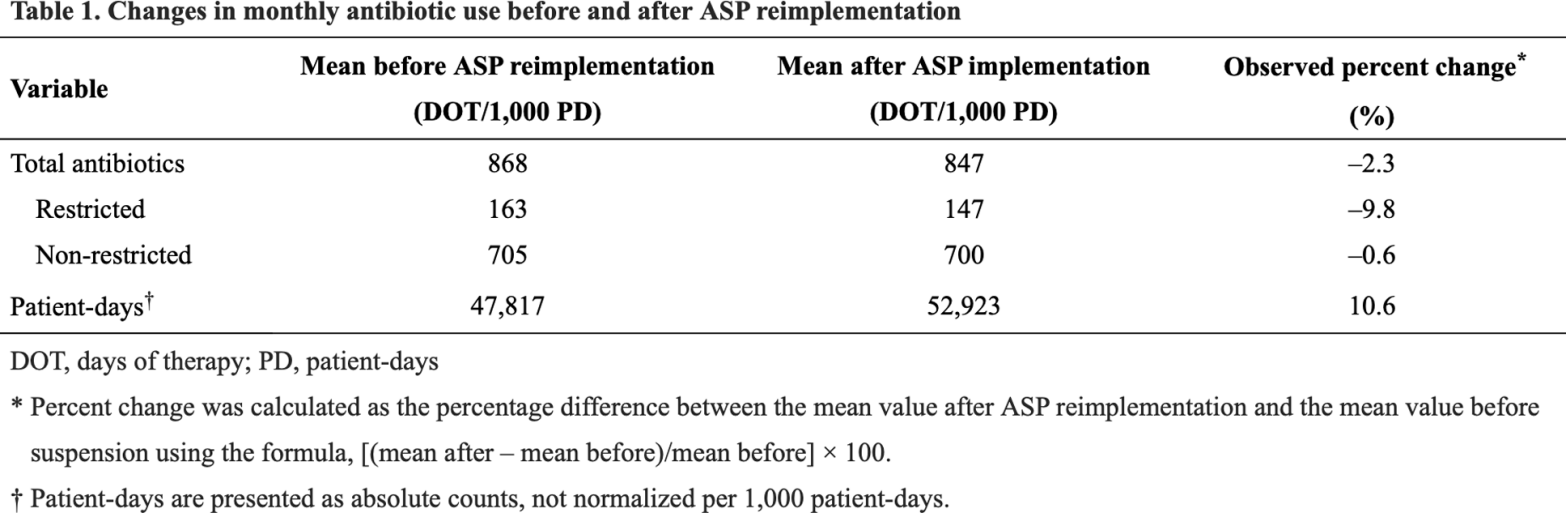

**Figure:**
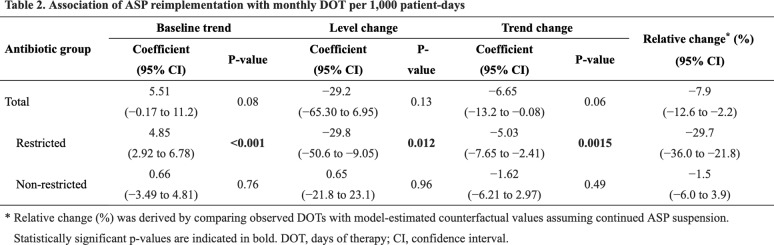

**Figure:**